# Association of iron deficiency anemia with tuberculosis in Taiwan: A nationwide population-based study

**DOI:** 10.1371/journal.pone.0221908

**Published:** 2019-08-30

**Authors:** Kuo-An Chu, Chun-Hsiang Hsu, Mei-Chen Lin, Yi-Hsin Chu, Yao-Min Hung, James Cheng-Chung Wei

**Affiliations:** 1 Division of Chest Medicine, Department of Internal Medicine, Kaohsiung Veterans General Hospital, Kaohsiung, Taiwan; 2 Department of Nursing, Shu-Zen Junior College of Medicine and Management, Kaohsiung, Taiwan; 3 School of Medicine, National Yang Ming University, Taipei, Taiwan; 4 Management Office for Health Data, China Medical University Hospital, Taichung, Taiwan; 5 Department of Family Medicine, National Cheng Kung University Hospital, Tainan, Taiwan; 6 Kaohsiung Municipal United Hospital, Kaohsiung, Taiwan; 7 Department of Emergency Medicine, Kaohsiung Veterans General Hospital, Kaohsiung, Taiwan; 8 Yuhing Junior College of Health Care and Management, Kaohsiung, Taiwan; 9 Department of Rheumatology, BenQ Medical Center, The Affiliated BenQ Hospital of Nanjing Medical University, China; 10 Institute of Medicine, Chung Shan Medical University, Taichung, Taiwan; 11 Department of Medicine, Chung Shan Medical University Hospital, Taichung, Taiwan; 12 Graduate Institute of Integrated Medicine, China Medical University, Taichung, Taiwan; University of Mississippi Medical Center, UNITED STATES

## Abstract

**Background:**

Iron deficiency is associated with decreased cellular immunity, which may predispose patients with iron deficiency anemia (IDA) to increased risk of developing tuberculosis (TB). This study investigated the relationship between newly diagnosed IDA and TB infection in Taiwan.

**Methods:**

The study included data on 21,946 patients with incident IDA and 87,555 non-IDA controls from a national database covering the period 2000–2012. IDA and non-IDA subjects were matched 1:4 on age, gender, and index year. The follow-up period was defined as the time from the initial IDA diagnosis to the date of developing TB or 31 December 2013. Cox proportional hazards models were used to estimate hazard ratios and 95% confidence intervals, with the control group as the reference.

**Results:**

The adjusted hazard ratio of TB for the IDA group was 1.99 (95% confidence interval, 1.77–2.25) compared with the control group. The subgroup analysis showed that for both genders, all age groups, and patients with diabetes mellitus, hyperlipidemia, hypertension, cancer, chronic obstructive pulmonary disease, and hepatitis B virus infection, the IDA group had significantly higher TB incidence. The association was significantly stronger within the 5 years after new IDA diagnosis for both genders and all age groups.

**Conclusions:**

Higher TB incidence was discovered in the IDA group, especially for patients with comorbidities.

## Introduction

Tuberculosis (TB), an infectious disease caused by *Mycobacteria tuberculosis*, is characterized by tubercle bacilli displaying intracellular survival strategies and chronic pulmonary inflammation [1]. The global burden of TB is still increasing, and the morbidity and mortality of TB remain substantial [2]. In 2012, approximately 8.6 million new cases of TB and an estimated 1.3 million related mortalities were reported globally [3]. TB affected approximately 10.4 million people and caused 1.7 million deaths worldwide in 2016 [4]. Preventing TB infection is thus a crucial global health issue. TB is endemic and highly prevalent in Taiwan. In 2012, the incidences of TB and TB-related death were 53 and 2.7 cases per 100,000 individuals, respectively [5]. Researchers have been searching for more means of preventing new cases of TB [2]. An assessment of potentially modifiable risk factors is a promising consideration for the formulation of TB control policies [6]. Some TB risk factors have been known for decades, including systemic diseases such as diabetes mellitus (DM) [7] and chronic kidney disease [8] as well as tobacco smoking [9], alcohol use [10], body mass index [11], silicosis [12], human immunodeficiency virus (HIV) infection [13], splenectomy [14], and gastrectomy [15,16]. Most of these risk factors can impair the human immune system, thereby increasing the risk of TB. However, few studies have investigated the link between nutritional iron deficiency and high TB prevalence.

Micronutrient deficiencies are a well-known global health threat, and poor nutritional status may predispose an individual to some infectious diseases [17]. Anemia, a critical global health problem, is the most common micronutrient deficiency, occurring in approximately one quarter of the world’s population [17,18]. Iron deficiency anemia (IDA) is the most significant contributor, accounting for 50% of all cases of anemia. IDA has a prevalence of 2%–5% among adult men and postmenopausal women in the developed world [19]. Animal and human studies have demonstrated that nutritional iron deficiency is associated with impaired phytohemagglutinin-induced lymphocyte proliferation and delayed-type hypersensitivity responses with relative preservation of humoral immunity [20–25].

TB and IDA are major public health concerns worldwide. Nonetheless, the relationship between iron deficiency, especially IDA, and the risk of contracting TB remains unclear. By using data in the Taiwan National Health Insurance Research Database (NHIRD) for the period January 1, 2000, to December 31, 2012 in the current population-based cohort study, we examined the association between newly diagnosed IDA and subsequent TB development.

## Material and methods

### Study design

This study is a retrospective matched-cohort study analyzing data over a period of 12 years in a nationwide, population-based database in Taiwan.

### Database

The IDA and control cohorts were created using the Taiwan NHIRD, which is maintained by a single national health insurance (NHI) program that covered 99.6% of Taiwan’s population until 2011. The NHIRD contains patient demographic information, encrypted identification number, gender, birth date, diagnostic data and procedures, all types of medical visits (outpatient department, emergency care, and hospitalizations), traditional Chinese medical services, and prescription drug use. The diagnostic and procedure codes are based on the International Classification of Diseases, Ninth Revision, Clinical Modification (ICD-9-CM). The Longitudinal Health Insurance Database 2000 (LHID 2000), a subset of the NHIRD, was employed in this study. The LHID 2010 is composed of all original claims data of 1,000,000 randomly sampled beneficiaries of the NHI program. No significant differences in age distribution, gender, or health care cost were noted between the 1,000,000 people in the LHID 2010 and the individuals in the NHIRD.

### Study population

Cases of IDA and TB were identified from the NHIRD by using the corresponding ICD-9 codes 280.X and 011–018, respectively, for the period from January 2000 to December 2012. We first identified patients with newly diagnosed IDA from data concerning both outpatient and inpatient visits. IDA was defined through diagnostic ICD codes and procedure codes, including complete blood count tests and serum ferritin tests. The index date was defined as the first date of IDA diagnosis. For further ascertainment, only patients with at least one inpatient admission or three outpatient visits during the 1 year after IDA was first diagnosed were selected. We then excluded patients who had previous history of TB (ICD-9-CM 010.x to 018.x and anti-TB drugs for 2 months), those <20 years of age, and those who withdrew from the insurance program before the index date. The IDA group finally comprised 21,946 patients. The non-IDA control group was randomly selected from the patients who had never been diagnosed with IDA or TB (selected at a 1:4 ratio matched by age, gender, and index year). The non-IDA control group comprised 87,555 people. Individuals in both groups were tracked until a TB event, withdrawal from the NHI program, or the end of 2013, whichever occurred first.

### Covariables

Factors that might influence the incidence of TB—such as age, gender, income level, and comorbidities—were used as independent variables. We classified age into three groups: 20–39, 40–64, and ≥65 years. The comorbidities analyzed in this study were hypertension (ICD-9-CM codes 401–405), DM (ICD-9-CM code 250), hyperlipidemia (ICD-9-CM code 272), chronic obstructive pulmonary disease (COPD; ICD-9-CM codes 491, 492, 496), cancer (ICD-9-CM codes 140–208), chronic kidney disease (CKD) (ICD-9-CM code 585),alcoholic liver disease (ICD-9-CM 571.0, 571.1, 571.3), liver cirrhosis (ICD-9-CM code 571.4), hepatitis B (ICD-9-CM codes 070.2, 070.3, V02.61), hepatitis C (ICD-9-CM codes 070.41, 070.44, 070.51, 070.54, V02.62), HIV infection (ICD-9-CM code 042–044 795.8 V08), pneumoconiosis (ICD-9-CM code 042–044 795.8 V08), splenectomy (ICD-9-CM procedure code 41.5), partial gastrectomy (ICD-9-OP 43.5, 43.6, 43.7, 43.8, 43.81, 43.82, 43.89), and total gastrectomy (ICD-9-OP 43.91 and 43.99). Information on comorbidities was obtained by tracing all ambulatory medical care and inpatient records in the NHI database for the 2 years before the index date.

### Outcome measurement

Regarding outcomes, we focused on the development of *Mycobacterium tuberculosis* infection. Diagnosis of TB was identified using ICD-9-CM codes 010–018 in combination with the prescription of at least two anti-TB drugs within 6 months of TB diagnosis.

### Statistical analysis

Proportional differences in independent variables between the IDA and control cohorts were analyzed using the Pearson χ^2^ test. The incidence of TB was expressed as the number of newly diagnosed TB cases per 10,000 person-years. To assess the risk of subsequently developing TB, we performed Cox regression analysis to obtain the crude and adjusted hazards ratios (HRs) and 95% confidence intervals (CIs) for the case group compared with the control group. Cox regression models were adjusted for age, gender, and all comorbidities. Furthermore, we performed stratified analysis by calculating the HRs for the patients with IDA according to different subgroups. The significance level was set to a two-tailed p value of 0.05. All data analyses were performed using SAS^®^ (version 9.4; SAS Institute, Inc., Cary, NC, USA).

This study was approved by the Institutional Review Board of China Medical University (permit number: CMUH-104-REC2-115**-**R3). Because all data were used anonymously and fully deidentified before analysis, the need for informed consent was waived by the board.

## Results

Overall, 413 individuals in the IDA group (which comprised 21,946 patients) developed newly onset TB during the follow-up period. The longest follow-up period was 8 years. Overall, the crude TB infection rate, which is the crude HR (95% CI), was 1.94 (1.73–2.17) for the IDA group.

### Demographic characteristics and comorbidities of patients with newly diagnosed IDA and the comparison cohort in Taiwan during 2000–2012

Table 1 displays the clinical characteristics of the patients with and without IDA. IDA was more prevalent in women than men (72.3% vs. 27.7%). The mean age at time of IDA diagnosis was 53.6 years. Comorbidities were more common in the IDA group.

**Table 1:** Demographic characteristics and comorbidities of patients with newly diagnosed iron deficiency anemia in Taiwan during 2000–2012.

Characteristics	Total	Iron deficiency anemia		p value
		No n = 87,555	Yes n = 21,946	
	n	n (%) / mean ± SD	n (%) / mean ± SD	
**Gender**				
Female	79182	63313 (72.3)	15869 (72.3)	0.99
Male	30319	24242 (27.7)	6077 (27.7)	
**Age**				
20–39	27025	21620 (24.7)	5405 (24.63)	0.88
40–64	48615	38892 (44.42)	9723 (44.30)	
≥65	33861	27043 (30.89)	6818 (31.07)	
Mean (SD) ^a^		53.55±18.29	53.72±18.40	0.22
**Baseline comorbidity**				
Hypertension	37518	28550 (32.6)	8968 (40.9)	<0.001
Diabetes mellitus	18896	13776 (15.7)	5120 (23.3)	<0.001
Hyperlipidemia	25005	19250 (22.0)	5755 (26.2)	<0.001
Chronic kidney disease	3143	1616 (1.8)	1527 (7.0)	<0.001
Cancer	39862	29043 (33.2)	10819 (49.3)	<0.001
COPD	16293	12280 (14.0)	4013 (18.3)	<0.001
Alcoholic liver disease	900	479 (0.5)	421 (1.9)	<0.001
Liver cirrhosis	2071	1007 (1.2)	1064 (4.8)	<0.001
Hepatitis B	3294	2398 (2.7)	896 (4.1)	<0.001
Hepatitis C	1668	1075 (1.2)	593 (2.7)	<0.001
HIV infection	32	18 (0.0)	14 (0.1)	<0.001
Pneumoconiosis	1149	780 (0.9)	369 (1.7)	<0.001
Splenectomy	90	52 (0.1)	38 (0.2)	<0.001
Gastrectomy	360	187 (0.2)	173 (0.8)	<0.001

^a^ Chi-square test, t-test

### Risk factors of new-onset TB among the patients with IDA and comparison cohort

**Fig 1:**
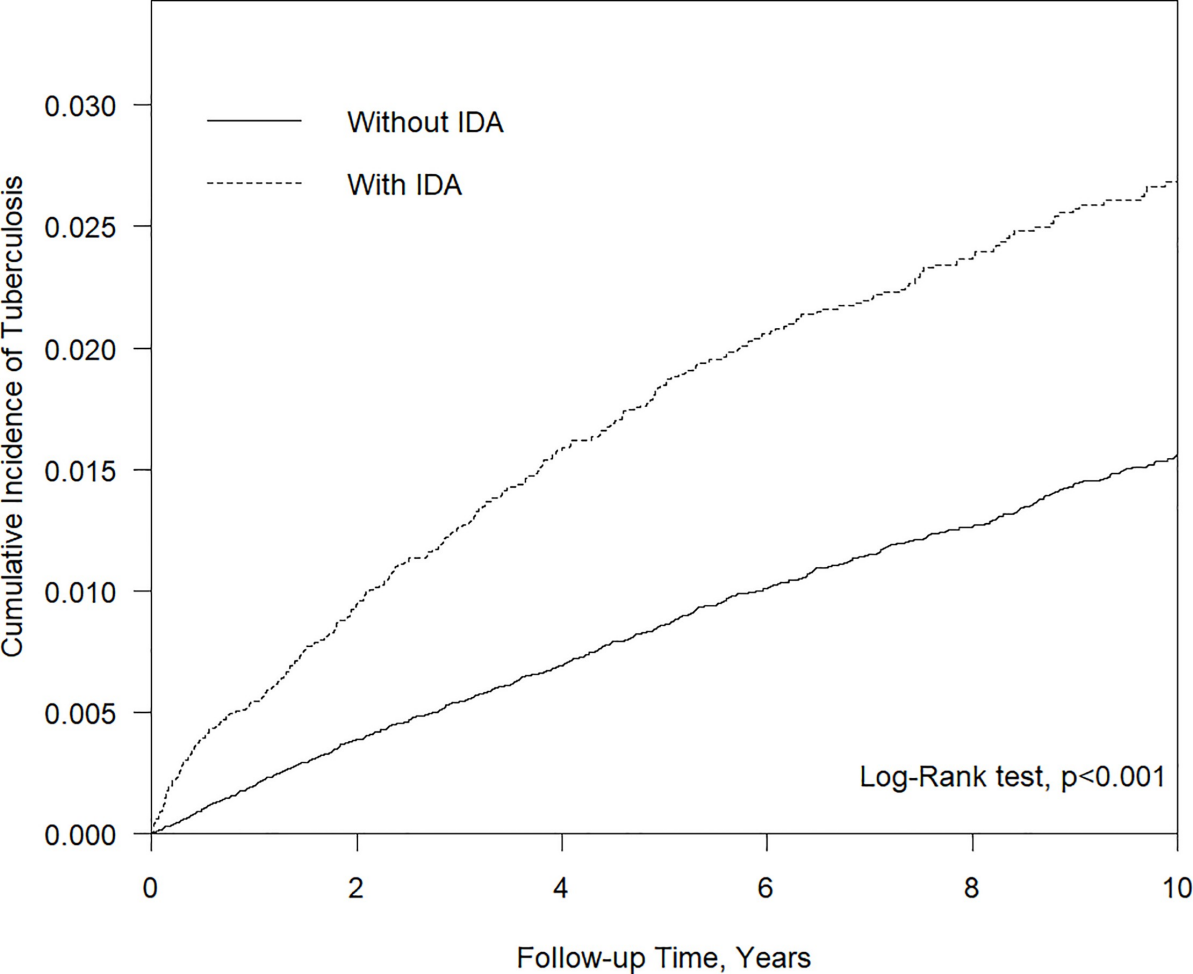
Cumulative incidence of TB in the IDA cohort and comparison group.

Table 2 presents the results of univariate and multivariate Cox regression analyses. Among all of the relevant variables, being in the IDA cohort, age > 40 years, male gender, gastrectomy, and comorbidities of DM, chronic kidney disease, COPD, pneumoconiosis, liver cirrhosis were the risk factors associated with a higher risk of developing TB. The crude HR (95% CI) for the IDA group was 1.94 (1.73–2.17). After adjustment for age, gender, and comorbidities, the risk of developing TB was similar (aHR, 1.99; 95% CI, 1.77–2.25). The survival curve (Fig 1) shows that the cumulative incidence of TB was higher in the IDA cohort than in the comparison group (p < 0.0001).

**Table 2:** Cox-model–measured hazard ratio and 95% confidence interval of tuberculosis associated with iron deficiency anemia in patients.

Characteristics	Event	Person	IR	Crude		Adjusted ^a^	
	(n = 1361)	year		HR (95% CI)	p value	HR (95% CI)	p value
**Iron deficiency anemia**							
No	948	589070	16.09	Ref.		Ref.	
Yes	413	131327	31.45	1.94(1.73–2.17)	<0.001	1.99(1.77–2.25)	<0.001
**Gender**							
Female	624	547074	11.41	Ref.		Ref.	
Male	737	173322	42.52	3.64(3.28–4.06)	<0.001	2.04(1.83–2.29)	<0.001
**Age at baseline**							
20–39	85	201705	4.21	Ref.		Ref.	
40–64	359	342640	10.48	2.48(1.95–3.14)	<0.001	2.21(1.74–2.81)	<0.001
≥65	917	176051	52.09	12.03(9.62–15.03)	<0.001	7.55(5.9–9.65)	<0.001
**Baseline comorbidity**							
Hypertension	773	207254	37.30	3.16(2.84–3.52)	<0.001	1.02(0.9–1.17)	0.75
Diabetes mellitus	398	100892	39.45	2.45(2.18–2.76)	<0.001	1.23(1.08–1.41)	0.002
Hyperlipidemia	398	141213	28.18	1.64(1.46–1.85)	<0.001	0.8(0.7–0.91)	<0.001
Chronic kidney disease	89	13095	67.96	3.54(2.85–4.39)	<0.001	1.38(1.1–1.72)	0.01
Cancer	441	240071	18.37	0.94(0.84–1.05)	0.27	0.89(0.79–1)	0.05
COPD	479	82567	58.01	4.05(3.62–4.53)	<0.001	1.75(1.55–1.98)	<0.001
Alcoholic liver disease	20	4254	47.02	2.38(1.53–3.70)	<0.001	1.38(0.87–2.17)	0.17
Liver cirrhosis	56	8448	66.29	3.38(2.58–4.42)	<0.001	1.53(1.15–2.05)	0.004
Hepatitis B	24	16922	14.18	0.71(0.48–1.07)	0.10	0.62(0.41–0.94)	0.02
Hepatitis C	34	7658	44.40	2.25(1.60–3.16)	<0.001	1.15(0.8–1.64)	0.46
HIV infection	1	139	71.79	3.54(0.50–25.00)	0.20	2.77(0.39–19.77)	0.31
Pneumoconiosis	43	4666	92.16	4.72(3.48–6.40)	<0.001	1.66(1.22–2.25)	0.001
Splenectomy	2	364	54.87	2.71(0.68–10.83)	0.16	1.34(0.33–5.44)	0.68
Gastrectomy	16	1699	94.15	4.81(2.94–7.88)	<0.001	1.74(1.05–2.87)	0.03

HR, hazard ratio; CI, confidence interval.

^a^ Adjusted HR: adjusted for gender, age, and all comorbidities in the Cox proportional hazards regression.

### Incidence rate, HR, and CI of TB in different subgroups

Table 3 reveals the association of risk of TB between gender, age, and comorbidity subgroups. In the gender-subgroup analysis, both men and women with IDA had higher TB risk than those without IDA. In all age groups, the IDA group had a higher risk of TB than the comparison group. In different stratifications of baseline comorbidities, the patients with DM, hyperlipidemia, hypertension, cancer, COPD, or hepatitis B virus infection in the IDA group had a significantly higher risk of TB. For the patients with hepatitis B virus infection, the IDA group had a 5.98-fold higher risk of TB (95% CI, 2.5–14.32).

**Table 3:** Incidence rates, hazard ratio, and confidence interval of tuberculosis in different stratifications.

Variables	Control			Case			Case VS. Control	
	n = 87,555			n = 21,946			Crude HR	Adjusted HR ^a^
	Event	Person years	IR	Event	Person years	IR	(95% CI)	(95% CI)
**Overall**	948	589070	16.09	413	131327	31.45	1.94(1.73–2.17) ^c^	1.99(1.77–2.25) ^c^
**Gender**								
Female	426	443558	9.60	198	103516	19.13	1.98(1.68–2.35) ^c^	1.96(1.64–2.33) ^c^
Male	522	145512	35.87	215	27811	77.31	2.11(1.80–2.47) ^c^	2.02(1.72–2.39) ^c^
**Age at baseline**								
20–39	48	161804	2.97	37	39901	9.27	3.12(2.03–4.80) ^c^	2.75(1.75–4.33) ^c^
40–64	228	278791	8.18	131	63850	20.52	2.50(2.02–3.11) ^c^	2.11(1.68–2.65) ^c^
≥65	672	148475	45.26	245	27576	88.84	1.93(1.66–2.23) ^c^	1.83(1.57–2.13) ^c^
**Baseline comorbidity**								
Hypertension	532	164495	32.34	241	42759	56.36	1.71(1.47–2.00) ^c^	1.76(1.51–2.06) ^c^
Diabetes mellitus	258	77158	33.44	140	23734	58.99	1.73(1.41–2.12) ^c^	1.77(1.43–2.19) ^c^
Hyperlipidemia	277	111779	24.78	121	29434	41.11	1.64(1.33–2.03) ^c^	1.67(1.34–2.08) ^c^
Chronic kidney disease	42	6846	61.35	47	6249	75.21	1.25(0.83–1.90)	1.34(0.87–2.06)
Cancer	285	178190	15.99	156	61881	25.21	1.57(1.30–1.91) ^c^	1.78(1.46–2.18) ^c^
COPD	335	64860	51.65	144	17707	81.32	1.56(1.28–1.89) ^c^	1.68(1.37–2.05) ^c^
Alcoholic liver disease	8	2507	31.91	12	1746	68.71	2.25(0.92–5.52)	2.09(0.79–5.52)
Liver cirrhosis	23	4650	49.46	33	3798	86.89	1.71(1.01–2.92) ^b^	1.73(0.99–3.04)
Hepatitis B	8	12777	6.26	16	4145	38.60	6.17(2.64–14.42) ^c^	5.98(2.5–14.32) ^c^
Hepatitis C	21	5272	39.83	13	2386	54.49	1.35(0.68–2.70)	1.42(0.69–2.91)
HIV infection	1	62	161.07	0	77	0.00	-	-
Pneumoconiosis	31	3567	86.90	12	1099	109.23	1.21(0.62–2.35)	1.20(0.60–2.40)
Splenectomy	0	247	0.00	2	117	170.89	-	-
Gastrectomy	6	870	68.95	10	829	120.59	1.77(0.64–4.88)	1.74(0.57–5.31)

IR, incidence rates, per 10,000 person-years; HR, hazard ratio; CI, confidence interval.

^a^ Adjusted HR: adjusted for gender, age, and all comorbidities in Cox proportional hazards regression.

^b^ p < 0.05

^c^ p < 0.001

### Risk of tuberculosis according to follow-up years by age group

Table 4 details the incidence and HR of TB stratified by follow-up years. In the analysis of the three age subgroups, the aHR (95% CI) of TB for the IDA group was the highest and most significant in the first 2 years, at 3.33 (1.61–6.88), 2.59 (1.75–3.84), and 2.15 (1.72–2.69) in the 20–39, 40–64, and ≥65 years age groups, respectively (p < 0.001). In all age subgroups, IDA was associated with higher incidence of subsequent TB, especially within the first 2 years after IDA diagnosis; even 5 years after IDA diagnosis, these patients were more likely to develop TB. In the 40–64 years age subgroup, the patients with IDA exhibited a significantly increased association with TB compared with the comparison group even after ≥5 follow-up years.

**Table 4:** Incidence and hazard ratio of tuberculosis with stratification by follow-up year.

Variables	Control			Case			Case VS. Control	
	n = 87,555			n = 21,946			Crude HR	Adjusted HR ^a^
	Event	Person years	IR	Event	Person years	IR	(95% CI)	(95% CI)
**Patients less than aged 40 years**								
<2	17	42128	4.04	16	10454	15.30	3.80(1.92–7.51) ^d^	3.33(1.61–6.88) ^c^
2–5	10	51149	1.96	8	12585	6.36	3.25(1.28–8.24) ^b^	3.01(1.14–7.97) ^b^
≥5	21	68527	3.06	13	16862	7.71	2.52(1.26–5.03) ^c^	1.98(0.93–4.19)
**Patients aged 40–64 years**								
<2	62	75810	8.18	52	18173	28.61	3.50(2.42–5.06) ^d^	2.59(1.75–3.84) ^d^
2–5	74	90030	8.22	46	20757	22.16	2.70(1.87–3.91) ^d^	2.34(1.58–3.47) ^d^
≥5	92	112750	8.16	33	24919	13.24	1.63(1.10–2.43) ^b^	1.57(1.04–2.36) ^b^
**Patients more than aged 65 years**								
<2	246	50040	49.16	122	10807	112.89	2.27(1.83–2.82) ^d^	2.15(1.72–2.69) ^d^
2–5	221	51345	43.04	77	9370	82.18	1.91(1.47–2.48) ^d^	1.81(1.39–2.36) ^d^
≥5	205	47090	43.53	46	7400	62.16	1.42(1.03–1.96) ^b^	1.35(0.97–1.87)

IR, incidence rates, per 10,000 person-years; HR, hazard ratio; CI, confidence interval.

^a^ Adjusted HR: adjusted for gender, age, and all comorbidities in Cox proportional hazards regression.

^b^ p < 0.05

^c^ p < 0.01

^d^ p < 0.001

## Discussion

In this nationwide population-based study of data covering 12 years, we discovered that people with new diagnoses of IDA were nearly twice as likely to subsequently develop TB than were those without IDA. Overall, IDA was associated with a 99% increased incidence of TB compared with the matched group. This result supports the hypothesis that individuals with iron deficiency are more susceptible to infections, perhaps because of impaired cell-mediated immunity. Furthermore, the effects of IDA were found to be more significant in some high-risk groups, such as patients with DM, hyperlipidemia, hypertension, cancer, COPD, or hepatitis B virus infection. Age is also another consideration for the prevention of TB among those with newly diagnosed IDA. Age had effects on both the strength and duration of the TB association.

To the best of our knowledge, this is the first large-scale study to assess the association of newly diagnosed IDA with subsequent TB. Our study is unique for several reasons. First, the sample size of the current study was large (enrolling 109,501 patients overall) and the follow-up period (131,326.7 person-years for the IDA cohort) was longer than in all other studies investigating the incidence of TB for various subgroups of patients with anemia. This strength enabled us to analyze the association of IDA with TB in groups stratified by age, gender, comorbidities, and follow-up period. Second, our data were obtained from a national insurance database covering a whole country with a single ethnic population; thus, the results are superior to those in smaller, single-hospital, and specific age or gender studies and those using purposive sampling [26,27]. This strength minimized potential bias from the sampling process [28]. Third, we defined IDA and TB by using accurate diagnosis criteria. IDA cases were defined using both ICD codes and procedure codes, whereas TB diagnosis was defined using both ICD codes and prescription codes for at least two anti-TB drugs.

The study data revealed that compared with the general population, patients with IDA had a greater incidence of subsequent TB development. The overall TB risk of the patients with IDA was higher than that of general population (aHR, 1.99 [95% CI, 1.77–2.25]), with an aHR (95% CI) of 2.02 (1.72–2.39) and 1.96 (1.64–2.33) for men and women, respectively. In addition, we identified some high-TB-risk groups of patients with IDA. Patients with DM, hyperlipidemia, hypertension, cancer, COPD, or hepatitis B virus infection in the IDA group were significantly more likely to develop TB. For the patients with hepatitis B virus infection, the IDA group had a 5.98-fold higher association with TB (95% CI, 2.5–14.32).

Another finding of this study that deserves attention is the effect of age on the TB association and the duration of the association. For all age groups, the IDA group had a stronger association with TB than the comparison group. However, as age increased, the association with TB infection became weaker. The aHR (95% CI) of TB association decreased from 2.75 (1.75–4.33) in the 20–39 years subgroup to 1.83 (1.57–2.13) in the ≥65 years subgroup. In all age subgroups, IDA was associated with higher incidence of subsequent TB, especially in the first 2 years after IDA diagnosis. Even 5 years after IDA diagnosis, these patients were still more likely to develop TB. However, only in the 40–64 subgroup, patients with IDA exhibited a significantly higher association with TB than the comparison group even after ≥5 follow-up years.

Several reasons may explain why more young patients with IDA developed TB. First, younger people tend to exhibit more symptoms and thus may be more likely to receive a TB diagnosis. Second, it is more likely for older people to be lost to follow-up than younger people with IDA because several years may have passed since their IDA diagnosis. After several years of follow-up, they may have also been eating iron-rich foods or independently taking drugs rather than visiting a doctor to obtain such drugs. This may have resulted in older patients with IDA being less frequently recorded in the NHIRD database.

The present findings have both clinical and public health implications. Clinically, physicians and patients should be aware of the possible association between TB and IDA. When treating patients with IDA and DM, hyperlipidemia, hypertension, cancer, COPD, or hepatitis B virus infection, clinicians must be aware of the increased risk of TB incidence. From a public health perspective, policymakers can consider implementing a TB screening test for certain high-risk patients with IDA.

Some limitations should be noted. Determining a strong association between IDA and TB by using a diagnosis database is extremely difficult and potentially uncertain. First, the diagnoses of IDA and TB in this study were mainly based on diagnostic ICD codes from insurance claims data rather than medical record review, which may have caused misclassification bias. To improve diagnostic validity, IDA cases were identified through diagnostic ICD codes and procedure codes and TB cases through both diagnostic ICD codes and prescription codes. In Taiwan, diagnosis of anemia is primarily based on complete blood count; when doctors determine that the complete blood count reveals microcytic anemia, they order further laboratory testing on serum iron, ferritin, and TIBA. IDA diagnoses are made when serum ferritin levels are low. In addition, diagnosis of TB in Taiwan is based on culture and image findings but sometimes through tissue biopsy and pathological findings. Second, data on alcohol consumption, smoking, homosexual or bisexual behaviors, malnutrition, socioeconomic status, body mass index, and severity of iron deficiency are unavailable in the NHIRD and are all potential confounding factors of TB. Consequently, we could not adjust for these variables and conduct related analysis. To partially address this, we used COPD as a proxy variable for cigarette smoking, similar to some other studies [29–31]. Third, our longitudinal follow-up study demonstrated an association but not a causal relationship. Moreover, we could not determine whether the etiology, severity, and duration of IDA were related to the development of TB. Further studies concerning whether IDA severity is related to TB infection rate should be conducted. Finally, IDA is more common in women; thus, our sample is not representative of the global TB population; moreover, most Taiwanese people have Chinese ethnicity, and our findings therefore may not be generalizable to other racial groups. Our results, therefore, should be cautiously interpreted.

## Conclusion

This 12-year nationwide population-based cohort study determined that patients with newly diagnosed IDA had increased incidence of subsequent TB, regardless of gender and age. Age had effects on both the strength and duration of the TB association. Future studies are required to explore the mechanisms underlying these associations. Clinicians are suggested to be aware of the higher TB risk of patients with new IDA diagnosis and to provide appropriate monitoring of high-risk groups.
